# Effects of Doula Care on Mother and Infant Health Outcomes in Al-Ahsa Region, Saudi Arabia: A Retrospective Cohort Study

**DOI:** 10.7759/cureus.65235

**Published:** 2024-07-24

**Authors:** Sakinah S Al-Zahir, Rahma AlGadeeb, Sumaya AlGadeeb, Shaimaa S Al-Zahir, Bayan S Al-Zahir, Zahra J Alfaraj, Salman A Alalshaikh

**Affiliations:** 1 Preventive Health Department, Rural Health Network, Central Division, Eastern Health Cluster, Ministry of Health, Safwa, SAU; 2 Preventive Medicine Department, Al-Ahsa Health Cluster, Ministry of Health, Hofuf, SAU; 3 Therapeutic Services Department, Al-Ahsa Health Cluster, Ministry of Health, Hofuf, SAU; 4 Medicine and Surgery Department, Faculty of Medicine, University of Alexandria, Alexandria, EGY; 5 Operation and Anesthesia Department, Qatif Health Network, First Eastern Cluster, Ministry of Health, Qatif, SAU; 6 Laboratory Department, Qatif Health Network, First Eastern Cluster, Ministry of Health, Qatif, SAU; 7 Emergency Medical Services Department, Qatif Health Network, First Eastern Cluster, Ministry of Health, Qatif, SAU

**Keywords:** saudi arabia, mental health, delivery data, outcome, pregnancy, traditional care, doula care

## Abstract

Introduction

Continued supportive care during childbirth may be the key to preventing unfavorable outcomes for both mother and child. It is important to assess and comprehend the sources of assistance available during pregnancy in order to enhance the birthing process and promote favorable outcomes.

Objectives

The current study aimed to assess the impact of ongoing doula support on mother and infant health outcomes compared to standard care.

Methods

A retrospective cohort study using both medical records and direct interviews based on information in the data collection form was carried out in the Eastern Province of Saudi Arabia, Al-Ahsa Region. The data that were collected included demographic information, obstetric history, delivery data, and postpartum outcome.

Results

A total of 50 pregnant women receiving doula care and 100 pregnant women receiving standard care were included. Regarding the mode of delivery, 43 (86%) of the doula group had vaginal deliveries compared to 73 (73%) of the standard care group, while CSs were needed for seven (14%) and 27 (27%), respectively (P = 0.78). Only seven (14%) neonates in the doula group required neonatal intensive care unit admission, versus 22 (22%) in the standard care group (P = 0.246). Additionally, initial breastfeeding within the golden hour occurred in 27 (54%) of the doula group compared to 16 (16%) of the standard care group, while exclusive breastfeeding was reported in 32 (64%) of the doula group compared to 26 (26%) of the standard care group (P < 0.001).

Conclusion

The current study showed more advantageous delivery and postpartum outcomes among doula care women and their infants compared to standard care, mainly for increasing the rate of initiated breastfeeding within the golden hour, exclusive breastfeeding, and reducing postpartum depression.

## Introduction

Women have traditionally received support in the hospital environment from female family members or partners as well as the nurses who are responsible for their care [1]. Patients place a great emphasis on the supportive role that nurses provide [2,3]. It is challenging for nurses to constantly support women and their spouses in the normal obstetric ward, though, given their varied tasks in intrapartum care [4]. In addition to the assistance of family members and hospital professionals, many women in labor opt to use doula services [5].

Doulas are trained professionals who assist women physically and emotionally during pregnancy, childbirth, and the postpartum period [6]. They are also known as birthing coaches [7]. Throughout the whole pregnancy and delivery process, they offer continuous assistance. Women who are in labor may benefit from one-on-one support in addition to nursing care [8]. It has been demonstrated that doulas and other support workers have a positive impact on women giving birth [9]. Doulas do not make decisions for mothers. Instead, they advocate on behalf of the patient to ensure that other healthcare professionals follow the patient's birth plan, if possible, and encourage the pregnant woman to ask questions at appointments and voice any worries or preferences [10].

Professional doulas attend to the physical, social, and emotional needs of the woman during pregnancy, labor, and delivery; maternity care providers prioritize caring for the laboring woman's overall health as well as making safe, effective decisions regarding childbearing management [11]. Doulas may help the laboring woman learn breathing techniques, relaxation techniques, encouraging mobility, massage, and position changes. They may also assist women and their families in anticipating labor events and may help a woman communicate with her maternity care provider during labor [6,12]. In the postpartum setting, doulas may provide breastfeeding support, emotional and physical postpartum recovery, coping strategies, and appropriate referrals [13]. The objective of the present study was to ascertain the influence of continuous doula care on mother and infant health outcomes in comparison to conventional standard care.

## Materials and methods

A retrospective cohort study, using both medical records and direct interviews based on information from the data collection form prepared from a comprehensive literature review, was carried out for women with doula support during pregnancy and women with standard care who gave birth between 2022 and 2023 in the Eastern Province of Saudi Arabia, Al-Ahsa Region. Al-Ahsa is the largest region in the Eastern Province.

Assisting Mothers for Active, Natural, and Instinctive Birth (AMANI Birth) [14], an Islamically based childbirth education and doula program, is dedicated to empowering and supporting women in making informed decisions about their birth. AMANI Birth has been registered as a doula and health education provider in the United States since September 7th, 2012. There are seven certified AMANI Birth doulas in Al-Ahsa. All pregnant women who received support from these doulas and gave birth between 2022 and 2023 were invited to participate in the study as the exposure group. The control group consisted of women who received standard care. The control group was selected from the Well Baby Clinic in the primary healthcare centers based on matching by residency. In Al-Ahsa, primary healthcare centers were divided into four sectors (clusters), designated as the northern, southern, middle, and eastern sectors, with a total of 53 primary healthcare centers. The distribution of primary healthcare centers among sectors is relatively homogeneous, with approximately 13 centers per sector. The participants of the control group (mothers with children aged between two months and two years) were selected by simple random sampling from the Well Baby Clinic in each matched primary healthcare center.

Assuming a 50% risk reduction with doula care, the study aimed for a power of 80% and an alpha error of 0.05. The minimum required sample size was 50 per group. The study matched exposed (doula-supported) women with women receiving standard care in a 1:2 ratio based on residency, necessitating 100 women in the standard care group. Thus, the total sample size was 150 women.

Saudi women with singleton pregnancies, aged 18-40 years, who delivered with the assistance of a doula or received standard care and were residents of Al-Ahsa between 2022 and 2023, were included in the study. Conversely, those with high-risk pregnancies, such as those involving smoking during pregnancy, elective CS delivery, chronic diseases (diabetes, hypertension, heart disease, mental illness, epilepsy, pulmonary disease), or maternal complications like gestational diabetes and pregnancy-induced hypertension, were excluded.

The study groups of pregnant women were interviewed to secure information on birth outcomes. Arabic-speaking interviewers, who were briefed about the study and trained in data collection, conducted the interviews. Obstetricians and gynecologists assessed the validity of the data collection sheet, and its reliability was measured using Cronbach’s alpha (0.72) for internal consistency.

The data collected included demographic information such as maternal age, income, education level, residency, and employment status. Other information collected from medical records included the type of birth (vaginal or CS), length of labor, low birth weight, and history of complications at birth for either the mother or baby. Additionally, data on the postpartum period, including postpartum depression (PPD), type of infant feeding, initiation of breastfeeding, and duration of exclusive breastfeeding, were collected.

The health outcomes included anemia, defined as hemoglobin levels <12.0 g/dL in women [15]; mode of delivery, either vaginal or CS delivery; PPD, a type of depression that occurs after childbirth and may persist for more than two weeks, as defined in the Diagnostic and Statistical Manual of Mental Disorders, Fifth Edition (DSM-5) [16], and confirmed by clinical assessment presented in the medical file; initial breastfeeding, the time when mothers begin providing breast milk to infants; the golden hour, the first hour after birth; exclusive breastfeeding, receiving only breast milk for the first six months of life; low birth weight, defined as weighing less than 2500 g at birth; and neonatal intensive care unit (NICU) admission for ill or preterm infants.

Data analysis

The data were collected, reviewed, and then fed to SPSS Statistics version 26 (IBM Corp. Released 2019. IBM SPSS Statistics for Windows, Version 26.0. Armonk, NY: IBM Corp). All statistical methods used were two-tailed with an alpha level of 0.05, considering significance if the P-value is less than or equal to 0.05. Descriptive analysis was done by prescribing frequency distribution and percentage for study variables among the two groups of mothers (doula care versus standard care group), including personal data, obstetric history, delivery data, and postpartum data. All comparisons were done using Pearson's chi-square test and the exact probability test for small frequency distributions. Logistic regression was done to adjust for some potential confounders: level of education, occupation, parity, and previous breastfeeding.

Ethical considerations

For the purpose of confidentiality, it is assured that all information provided will be treated with strict confidentiality and used solely for research purposes. Informed consent was obtained from primary healthcare providers and hospital administration after the study's approval. The study received ethical approval from the King Fahad Hospital - Hofuf Institutional Review Board (approval number: 12-E-2023).

## Results

A total of 50 pregnant women receiving doula care and 100 pregnant women receiving standard care were included. In terms of the sector of care, the most common among both groups was the middle sector (46% for each) and then the eastern sector (30% for each). Regarding age, the mean age of the doula care group was 29.1 ± 5.0 years, compared to 30.0 ± 5.6 years for the standard care group, with no statistical significance (P = 0.351). Concerning educational level, 76% of the doula care group had a university level of education, compared to 54% of the standard care group, with a recorded statistical significance (P = 0.006). Among the doula care group, 33 women (66%) were housewives, while 16 women (32%) were working, compared to 76 women (76%) and 18 women (18%) in the standard care group, respectively (P = 0.166). A monthly income of less than 5000 SR was reported by five women (10%) in the doula care group, compared to 26 women (26%) in the standard care group, while a monthly income of 11,000 to 20,000 SR was reported by 24 women (48%) in the doula care group and 24 women (24%) in the standard care group (P = 0.008) (Table 1).

**Table 1:** Maternal Characteristics of the Study Groups. SD: standard deviation, SR: Saudi Riyal ^: exact probability test, *: significant at P < 0.05

Characteristics	Doula (N = 50)	Standard (N = 100)	P-value
Age, mean ± SD	29.1 ± 5.0	30.0 ± 5.6	0.351
Residency sector, n (%)			
Eastern	15 (30.0%)	30 (30.0%)	1.0
Middle	23 (46.0%)	46 (46.0%)	
Northern	9 (18.0%)	18 (18.0%)	
Southern	3 (6.0%)	6 (6.0%)	
Education level, n (%)			
Secondary/less	6 (12.0%)	37 (37.0)	0.006*
University	38 (76.0%)	54 (54.0%)	
Higher education	6 (12.0%)	9 (9.0%)	
Occupation, n (%)			
Housewife	33 (66.0%)	76 (76.0%)	0.166^
Student	1 (2.0%)	6 (6.0%)	
Employee	16 (32.0%)	18 (18.0%)	
Monthly income, n (%)			
<5,000 SR	5 (10.0%)	26 (26.0%)	0.008*
5,000-1,0000 SR	15 (30.0%)	42 (42.0%)	
11,000-20,000 SR	24 (48.0%)	24 (24.0%)	
>20,000 SR	6 (12.0%)	8 (8.0%)	
Parity, n (%)			
Nulliparous	15 (30.0%)	22 (22.0%)	0.740
2 times	12 (24.0%)	23 (23.0%)	
3 times	12 (24.0%)	25 (25.0%)	
4 times	7 (14.0%)	16 (16.0%)	
Grand multipara (≥ 5)	4 (8.0%)	14 (14.0%)	
History of abortion, n (%)			
Yes	10 (20.0%)	25 (25.0%)	0.495
No	40 (80.0%)	75 (75.0%)	
History of previous breastfeeding			
Yes	26 (52.0%)	71 (71.0%)	0.018*^
No	24 (48.0%)	29 (29.0%)	

**Figure 1:**
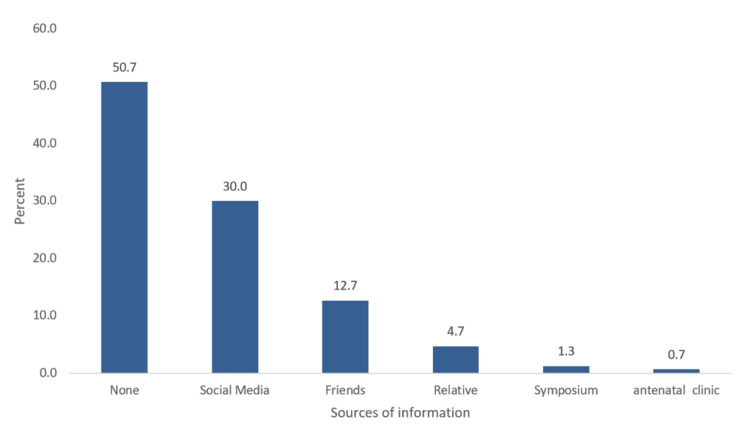
Sources of information about doula care

For the past obstetric history among study groups of pregnant women, regarding parity, 15 (30%) of women receiving doula care were nulliparous, compared to 22 (22%) in the standard care group. Additionally, four (8%) of the doula care group were grand multipara (≥5) compared to 14 (14%) in the standard care group (P = 0.740). A total of 10 (20%) in the doula care group had a history of previous abortions versus 25 (25%) in the standard care group (P = 0.495). Previous breastfeeding history was reported by 26 (74.3%) of the doula care group, compared to 71 (91%) in the standard care group (P = 0.018). Regarding the source of information about doula care, the most common were social media (30%), friends (12.7%), relatives (4.7%), symposiums (1.3%), and antenatal care clinics (0.7%), while about half of the participants were unfamiliar with doula care (Figure 1).

For maternal health outcomes, regarding anemia, six (12%) of the doula care group had anemia compared to 21 (21%) in the other group (P = 0.176). Regarding the mode of delivery, 43 (86%) of the doula group had vaginal deliveries compared to 73 (73%) in the standard care group, while CSs were needed for seven (14%) and 27 (27%) of each group, respectively (P = 0.78). The length of labor ranged from one to 72 hours in the doula group compared to one to 96 hours in the standard care group, with a median duration of 12 hours for both groups (P = 0.092). Women receiving doula care had significantly lower rates of PPD, with three (6.0%) compared to 27 (27.0%) in the standard care group (P = 0.002).

For infant health outcomes, babies born to women who received continuous support from a doula were less likely to have low birth weight (8.0%) compared to babies born to women who received standard care (12.0%), though this difference was not statistically significant (P = 0.42). Regarding NICU admission, seven (14.0%) neonates in the doula group required NICU admission versus 22 (22%) in the standard care group (P = 0.246). Initial breastfeeding within the golden hour occurred among 27 (54%) of mothers in the doula group compared to 16 (16%) in the standard care group, which was statistically significant (P = 0.001). Additionally, exclusive breastfeeding for six months or more was observed among 32 (64%) of doula group mothers compared to 26 (26%) of standard care mothers, which was also statistically significant (P = 0.001) (Table 2).

**Table 2:** Health outcomes of the study groups NICU: neonatal intensive care unit, CS: cesarean section, PPD: postpartum depression, ^: exact probability test, *: significant at P < 0.05

Health outcome	Doula (N = 50)	Standard (N = 100)	P-value
Maternal health outcomes, n (%)			
Anemia	6 (12.0%)	21 (21.0%)	0.176
CS delivery	7 (14.0%)	27 (27.0%)	0.78
Length of labor time, hour, median (range)	12 (1 - 72)	12 (1 - 96)	0.092
PPD	3 (6.0%)	(27.0%)	0.002*
Infant health outcome, n (%)			
Low birth weight	4 (8.0%)	12 (12.0%)	0.42
NICU	7 (14.0%)	22 (22.0%)	0.246
Breastfeeding within the golden hour	27 (54.0%)	16 (16.0%)	<0.001*^
Exclusive breastfeeding	32 (64.0%)	26 (26.0%)	<0.001*^

The study findings indicate that women who received doula care exhibited significantly lower odds of PPD compared to those who received standard care. The unadjusted odds ratio (OR) was 0.17 (95% confidence interval (CI): 0.05-0.60, P = 0.006), while the adjusted OR was 0.14 (95% CI: 0.03-0.64, P = 0.011). In addition, there was a significant increase in the proportion of women who initiated breastfeeding within the golden hour, with an unadjusted OR of 6.163 (95% CI: 2.849-13.330, P < 0.001) and an adjusted OR of 4.136 (95% CI: 1.556-10.994, P = 0.004). Furthermore, the rates of exclusive breastfeeding also demonstrated a significant increase, with an unadjusted OR of 5.060 (95% CI: 2.438-10.501, P < 0.001) and an adjusted OR of 5.93 (95% CI: 2.219-15.852, P < 0.001). However, no statistically significant differences were observed between the doula care and standard care groups for outcomes such as anemia, CS delivery, low birth weight, and NICU admission (all P > 0.05) (Table 3).

**Table 3:** ORs for health outcomes Logistic regression was used to adjust for some potential confounders. Anemia was adjusted for education level and income. CS delivery was adjusted for parity, age, education level, and length of labor. Post-partum depression was adjusted for age, education level, occupation, NICU, income, parity, and mode of delivery. Low birth weight was adjusted for age, parity, anemia, and education level. NICU admission was adjusted for low birth weight, length of labor, and mode of delivery. Breastfeeding and exclusive breastfeeding were adjusted for education level, mode of delivery, NICU, parity, previous breastfeeding, occupation, and income. ORs: odds ratios, CS: cesarean section, NICU: neonatal intensive care unit, *: P < 0.05 (significant)

Health outcome	Unadjusted OR (95% CI)	P-value	Adjusted OR (95% CI)	P-value
Anemia	0.513 (0.193-1.366)	0.182	0.496 (0.175-1.402)	0.186
CS delivery	0.440 (0.177-1.096)	0.78	0.442 (0.128-1.531)	0.198
Post-partum depression	0.17 (0.05-0.60)	0.006*	0.14 (0.03-0.64)	0.011*
Low birth weight	0.638 (0.195-2.089)	0.457	0.639 (0.189-2.16)	0.471
NICU	0.577 (0.228-1.461)	0.246	0.733 (0.269-1.99)	0.544
Breastfeeding within the golden hour	6.163 (2.849-13.330)	<0.001*	4.136 (1.556-10.994)	0.004*
Exclusive breastfeeding	5.060 (2.438-10.501)	<0.001*	5.93 (2.219-15.852)	<0.001*

## Discussion

Since maternal health is one of the world’s health priorities, increasing awareness of the role of doulas could be a key factor in achieving the Maternal Health Goals by 2030 [17]. While there is evidence supporting the use of one-on-one support care from previous literature reviews, the implementation of doula care remains controversial [18]. This controversy is due to the insufficient use of doulas. Unfortunately, we were unable to find any research in our community or in Arab countries on the importance and impact of doula care. Therefore, the current study assumes that pregnancy outcomes in pregnant women receiving doula care differ from those receiving standard care, with a positive effect on breastfeeding initiation and postpartum outcomes.

The current study revealed that both study groups were similar in all personal characteristics except for education and monthly income, which were somewhat better among the doula group. Additionally, past obstetric history was similar between the two groups, except that the history of previous breastfeeding was significantly higher among the standard care group of pregnant women. This may be due to the fact that the occupation level was higher among the doula group of females, which implies a higher likelihood of using artificial feeding due to work hours.

Considering delivery data, about 86% of women in the doula group had normal vaginal delivery, compared to 73% in the standard care group. Women in the doula group had a CS due to unavoidable causes such as failure to progress or premature rupture of membranes. These findings suggest that doula care reduced the rate of CSs due to avoidable causes and also had a minimal impact on reducing the length of labor. A study by Chen and Lee [19] reported a significant difference in natural childbirth rates (87.0% vs. 56.8%) and CS birth rates (13.0% vs. 43.2%) between the doula and control groups. A previous Cochrane analysis indicated that higher spontaneous vaginal birth rates and decreased CS birth rates were best achieved with ongoing care. By helping women weigh the advantages and disadvantages of induction, reducing the need for pain medication during labor, and advocating for their needs, doulas can help laboring women reduce their risk of CS [20]. Similarly, Sobczak et al. [21] documented that doula support in perinatal care was associated with positive delivery outcomes, including reduced CSs, fewer premature deliveries, and shorter labor durations. Sanjaya et al. [22] reported that 60% of women who received doula care gave birth naturally, while 31 patients (31%) who had a CS and were assisted by a doula came next.

Regarding postpartum effects, the current study revealed that early initiation of breastfeeding was significantly higher among women who received doula care, with better breastfeeding habits (longer exclusive breastfeeding duration) and a markedly lower rate of PPD. These findings favor doula care over standard care for pregnant women and are consistent with conclusions from many other studies [20-22]. Doula care in underserved communities improves a variety of health outcomes for both mothers and babies, lowers healthcare costs, reduces CS rates, reduces maternal distress and depression, and improves communication between pregnant women and their healthcare providers [21]. Several studies have reported that doula care is linked to improved pregnancy outcomes and reduced costs compared to standard care [23-25]. Additionally, reduced induction rates among women receiving doula care may contribute to higher birth weights and decreased CS rates. This may result from improved readiness for childbirth before delivery, enhanced flexibility in positions throughout labor, and the coping mechanisms offered by doulas during labor [10,26]. Overall, doula support can be beneficial, providing both emotional and physical support during childbirth [27]. The results indicated reduced infant mortality rates and epidural use, as well as increased lactogenesis in patient populations utilizing doulas [28,29].

Strength and limitations

Fortunately, our study is considered the first in Saudi Arabia and the Arab countries to focus on the importance of doula care and its impact. Despite the significant findings of this study, some limitations should be acknowledged. The study did not consider the potential influence of cultural factors on the acceptance and efficacy of doula care. Furthermore, the study design was observational, which precludes establishing causal relationships between doula care and improved maternal and infant health outcomes. Randomized controlled trials would be necessary to confirm the effectiveness of doula care in different populations and to account for potential confounding factors more rigorously.

## Conclusions

The current study showed the advantages of doula care regarding intrapartum and postpartum mother and infant health outcomes, particularly in increasing the rate of breastfeeding initiated within the golden hour, promoting exclusive breastfeeding, and reducing PPD. The effectiveness of doula care, especially for at-risk groups, combined with the positive outcomes reported, supports its use as an affordable approach to improving maternal and infant health and reducing disparities in childbearing. However, the use of doulas is still controversial and is believed to be underutilized. Raising awareness about doulas could be an important factor in achieving maternal health goals by 2030. Further research in Arab countries is needed for a more comprehensive evaluation of the role of doula care on mother and infant health outcomes to assist decision-makers in the proper planning of antenatal care policies.
